# Mammography of suspicious calcifications among ductal carcinoma in situ and benign breast disease

**DOI:** 10.4102/sajr.v28i1.2852

**Published:** 2024-05-13

**Authors:** Wanrudee Lohitvisate, Chidsupang Kaeorat, Amolchaya Kwankua

**Affiliations:** 1 Department of Radiology, Faculty of Medicine, Thammasat University, Pathumthani, Thailand

**Keywords:** ductal carcinoma in situ, benign breast disease, microcalcification, mammography, imaging features

## Abstract

**Background:**

Most ductal carcinoma in situ (DCIS) lesions manifest early as calcifications, which could be benign or malignant. The classified group of suspicious calcifications among DCIS and benign breast disease is clinically important to early evaluate patient risk factors and plan treatment options.

**Objectives:**

To compare imaging features of suspicious calcifications between DCIS and benign breast disease.

**Method:**

A retrospective study of 101 suspicious calcifications was performed at Thammasat University Hospital from June 2011 to October 2020. The calcifications were surgically excised by mammography-guided wire localisation. The mammographic features of the suspicious calcifications were reviewed according to the fifth edition of the American College of Radiology Breast Imaging-Reporting and Data System lexicon. For comparing between two groups, the student *t*-test, Fisher’s exact test and Mann-Whitney U test were used for statistical analyses. The logistic regression analysis was calculated for DCIS prediction.

**Results:**

The pathologic results of all 101 suspicious calcifications were DCIS (30 cases) and benign breast disease (71 cases). Linear morphology and segmental distribution correlated significantly with DCIS (*p* = 0.003 and *p* = 0.024, respectively). After multivariable analysis, fine linear calcification still significantly elevated the risk of DCIS (odd ratios, 51.72 [95% confidence interval: 2.61, 1022.89], *p*-value of 0.01), however, the odds of predicting DCIS was not statistically significant different among any distribution.

**Conclusion:**

Ductal carcinoma in situ calcification has contrasting morphology and distribution features compared to benign breast disease. The calcification descriptor is considered an important implement for early diagnosis and distinguishes DCIS from other benign breast conditions.

**Contribution:**

Calcification descriptor is considered an important implement for early diagnosis and distinguishment of DCIS from other benign breast conditions.

## Introduction

Female breast cancer is the most common cancer and the leading cause of death worldwide, contributing to 6.9% of all cancer deaths.^1^ Ductal carcinoma in situ (DCIS) is scrutinised as a precursor lesion for developing invasive breast cancer with a relative risk of 8% – 11%.^2^ Most DCIS lesions are initially detected on screening mammography when they may manifest as microcalcifications.^3,4^ The early detection of DCIS could change disease prognosis and treatment options.^5^

Breast microcalcifications, which are the main presentations of asymptomatic breast malignancies, account for 50% – 75% of mammographic findings.^6,7,8^ Breast microcalcifications may represent diverse pathological processes including inflammation, infection, benign tumour and malignancy.^9^ Thus, differentiating benign from malignant calcifications is a diagnostic challenge. In order to standardise the description and classify lesions into a category indicating the probability of malignancy, ranging between 0% and 100%, the American College of Radiology Breast Imaging-Reporting and Data System (ACR BI-RADS) was launched in 1993 and subsequently revised.^10^ The system categorises the morphology and distribution of calcifications, defining them benign or malignant. The BI-RADS nomenclatures of suspicious calcifications are new or progression of a group of punctate, amorphous, fine pleomorphic, coarse heterogeneous or fine linear calcifications,^10^ with suspicious BI-RADS 4 and 5 classifications necessitating a tissue diagnosis. However, this system has limited predictive value, with false-positive biopsy rates for calcifications between 30% and 87%.^11,12,13,14^

The two principal techniques for biopsy of suspicious calcifications include mammography-guided wire localisation with surgical excision and stereotactic-guided percutaneous biopsy. Surgical excision may be more beneficial than stereotactic-guided percutaneous biopsy in several circumstances, as the entire lesion is removed and additional unexpected abnormal findings may be detected which may be a valuable tool in morphology and histopathology correlation.^15,16,17^

The purpose of this study was to compare the mammographic features of suspicious calcifications between DCIS and benign breast disease.

## Research methods and design

### Study population

The data of female patients with suspicious microcalcifications on digital mammography who underwent guided wire localisation with surgical excision (*n* = 121) at Thammasat University Hospital from 01 June 2011 to 30 October 2020 were collected and retrospectively reviewed.

We conducted both screening mammography and diagnostic mammography, which had various clinical manifestations. Mammography-guided wire localisation with surgical excision was performed until the end of 2020. Stereotactic breast biopsy commenced in 2021 as the first method to target the calcifications, followed by mammography-guided wire localisation with surgical excision for malignant results, high-risk results or failed stereotactic biopsies. Stereotactic breast biopsy was omitted in some difficult cases, where wire localisation was preferred.

Patients who had suspicious calcifications on digital mammography that were classified as BI-RADS 4 or 5, patients whose digital mammography images were available on the Pictures Archiving and Communications Systems (PACS) and patients whose medical records and pathological results were accessible on the information system of the Thammasat University Hospital were included. Excluded patients were those where the digital mammography images displayed other findings such as a mass or architectural distortion, and where the pathological report revealed other histopathological besides DCIS such as invasive ductal carcinoma. Of the initial 121 suspicious calcifications, 18 cases were excluded because of unavailable histopathology on the hospital electronic database, one case had combined suspicious calcifications and an associated mass and one case had a final histopathologic diagnosis of invasive ductal carcinoma.

The suspicious calcifications were classified as BI-RADS 4B for amorphous, coarse heterogeneous and fine pleomorphic calcifications that were upgraded to BI-RADS 4C for segmental distribution. The linear branching calcifications were BI-RADS 4C, upgraded to BI-RADS 5 in cases of new calcifications with a segmental distribution. The new group or increased number or extension of the punctate or round calcifications on follow-up mammographic images were classified as BI-RADS 4A, requiring a tissue diagnosis.^10^

### Imaging technique, processing and interpretation

Two standard mammographic views including craniocaudal (CC) and mediolateral oblique (MLO) views were obtained, using the digital technique Lorad Selenia (Hologic^®^) for all patients at our institution. Additional positional techniques such as spot magnification or spot compression were performed in selected cases.

The digital mammography images were retrospectively reviewed by two radiologists with 12 years and 11 years of experience in routine work. For results with a discrepancy, a final consensus was reached after discussion. The images were randomly selected and blinded to the histopathology results.

The mammography images were retrieved retrospectively from the PACS. The latest images prior to mammography-guided wire localisation with surgical excision were selected and reviewed. The data included side, site (locations and depth), morphology and distribution of the suspicious calcifications by using the fifth edition ACR BI-RADS lexicon. The mammographic features were identified as follows: (1) the breast parenchymal density was divided into almost entirely fat, scattered areas of fibroglandular density, heterogeneously dense or extremely dense; (2) the locations of calcifications were classified as upper inner, upper mid, upper outer, subareolar, mid inner, mid outer, lower inner, lower mid and lower outer regions; (3) the morphology of calcifications was defined as punctate or round, amorphous, coarse heterogeneous, fine pleomorphic and fine linear or branching and (4) the distribution of calcifications was described as diffuse, regional, grouped, segmental and linear.

Furthermore, all prior digital mammography images were accessed and identified. In the event that calcifications were seen before, the extension of calcification was recorded in millimeters (measuring the longest dimension of calcifications). If the previous mammography images, on the other hand, did not demonstrate the calcifications, the lesion size was recorded as zero millimeter initially. The time interval between the previous and the latest mammography prior to mammography-guided wire localisation with surgical excision was recorded, allowing growth rates per month to be calculated.

### Clinicopathological evaluation

The patient’s clinical information was collected from the electronic medical records comprising age at diagnosis, clinical presentation (screening, palpable mass, breast pain or nipple discharge), menstrual status and hormonal use.

The histopathological data were retrieved from the histopathological reports, which were separated into two groups: DCIS and benign breast disease. The DCIS calcifications were categorised based on histologic tumour grade, using the European classification that divided tumour grade based on nuclear atypia. The calcifications classified as negative for malignancy were identified as benign breast disease.

### Data analysis and statistics

The patient characteristics and histopathological results were rigorously reported by using the number (percentage) for categorical variables and means, standard deviations (s.d.) and range for continuous variables.

To compare the clinicopathological characteristics and the imaging features between DCIS and benign breast disease, we used the student *t*-test for the normally distributed data and Mann-Whitney *U* test for non-normally distributed continuous variables. The Chi-square test and Fisher’s exact test were used for the categorical variables. All statistical analyses were performed with *R* program (version 4.1.1, *R* foundation for Statistical Computing, Vienna, Austria), and statistical significance was indicated at a *p*-value less than 0.05.

With regard to the data analyses, when mammographic features were discovered to be statistically significant, logistic regression analysis was conducted to calculate the odds ratio (OR) with 95% confidence intervals (CI) for predicting DCIS probability as compared with benign breast disease. Odds ratios were contemplated to indicate statistical difference if the 95% CI excluded 1.0.

### Ethical considerations

Ethical clearance to conduct this study was obtained from the Thammasat University, Human Ethics Committee (project number: MTU-EC-RA-0-261/63, certificate of approval number: 021/2021).

## Results

### Patient characteristics and histopathological results

A total of 101 suspicious calcifications were eligible the our study. The final pathology results from wire localised excision were DCIS (*n* = 30) and benign breast disease (*n* = 71). The demographic data, clinical characteristics and histopathological results of patients with DCIS and benign breast disease are displayed in Table 1. There were no statistically significant differences in patient’s age, clinical presentation or hormonal use between the DCIS and benign breast disease groups.

**TABLE 1:** Demographics, clinical characteristics and histopathological results of patients.

Characteristics	DCIS (*n* = 30)					Benign (*n* = 71)					Total (*N* = 101)					*p*
	Mean	Range	s.d.	*n*	%	Mean	Range	s.d.	*n*	%	Mean	Range	s.d.	*n*	%	
**Age**	53.5	26–80	11.9	-	-	51.4	35–73	9.3	-	-	52.1	26–80	10.2	-	-	0.252
**Clinical presentation**	-	-	-	-	-	-	-	-	-	-	-	-	-	-	-	0.099
Screening	-	-	-	19	63.3	-	-	-	49	69.0	-	-	-	68	67.3	-
Palpable mass	-	-	-	5	16.7	-	-	-	18	25.4	-	-	-	23	22.8	-
Breast pain	-	-	-	5	16.7	-	-	-	4	5.6	-	-	-	9	8.9	-
Nipple discharge	-	-	-	1	3.3	-	-	-	0	0	-	-	-	1	1.0	-
**Menstrual status**	-	-	-			-	-	-			-	-	-			0.01
Pre-menopause	-	-	-	9	30.0	-	-	-	36	50.7	-	-	-	45	44.6	-
Menopause	-	-	-	18	60.0	-	-	-	20	28.2	-	-	-	38	37.6	-
Unknown	-	-	-	3	10.0	-	-	-	15	21.1	-	-	-	18	17.8	-
**Hormonal used**	-	-	-			-	-	-			-	-	-			0.682
No	-	-	-	15	50.0	-	-	-	29	40.8	-	-	-	44	43.6	-
Yes	-	-	-	4	13.3	-	-	-	10	14.1	-	-	-	14	13.9	-
Unknown	-	-	-	11	36.7	-	-	-	32	45.1	-	-	-	43	42.6	-
**Histopathological report**	-	-	-			-	-	-	-	-	-	-	-	-	-	-
DCIS grade 1	-	-	-	4	13.3	-	-	-	-	-	-	-	-	-	-	-
DCIS grade 2	-	-	-	16	53.3	-	-	-	-	-	-	-	-	-	-	-
DCIS grade 3	-	-	-	10	33.3	-	-	-	-	-	-	-	-	-	-	-
Fibrocystic change	-	-	-	-	-	-	-	-	33	46.5	-	-	-	-	-	-
Atypical ductal hyperplasia	-	-	-	-	-	-	-	-	9	12.7	-	-	-	-	-	-
Sclerosing adenosis	-	-	-	-	-	-	-	-	9	12.7	-	-	-	-	-	-
Fat necrosis	-	-	-	-	-	-	-	-	3	4.2	-	-	-	-	-	-
Radial scar	-	-	-	-	-	-	-	-	2	2.8	-	-	-	-	-	-
Fibroadenoma	-	-	-	-	-	-	-	-	2	2.8	-	-	-	-	-	-
Fibroadenomatous hyperplasia	-	-	-	-	-	-	-	-	1	1.4	-	-	-	-	-	-
Others†	-	-	-		-	-	-	-	-	8.5		-	-	-	-	-

DCIS, ductal carcinoma in situ; s.d., standard deviation.

† , Others included columnar cell change and hyperplasia (*n* = 2), foci of calcification without malignancy (*n* = 1), focal fibrotic stroma (*n* = 1), fibroepithelial lesion (*n* = 1), breast hyalinised stroma and duct ectasia (*n* = 1).

### Mammographic features

No significant differences in distribution of breast density, site, location or depth of lesions were detected between DCIS and benign breast disease. Heterogeneously dense fibroglandular tissue was the most common breast density in both groups (73.3% for the DCIS group and 70.4% for the benign group). The most common lesion location was the upper outer quadrant: 40% for the DCIS group and 54.9% for the benign group.

The calcifications were classified into four groups (BI-RADS 4A, 4B, 4C and 5 categories). Fourteen cases revealed BIRADS 4A calcifications, of which 12 (85.7%) were benign and 2 (14.2%) were DCIS. In 65 cases, the calcifications were BIRADS 4B: benign in 48 (73.9%) and DCIS in 17 (26.2%). Twenty cases of BI-RADS 4C calcifications were benign in 11 (55%) and DCIS in 9 (45%). Only two cases, both of which were DCIS, were classified as BIRADS 5.

**FIGURE 1:**
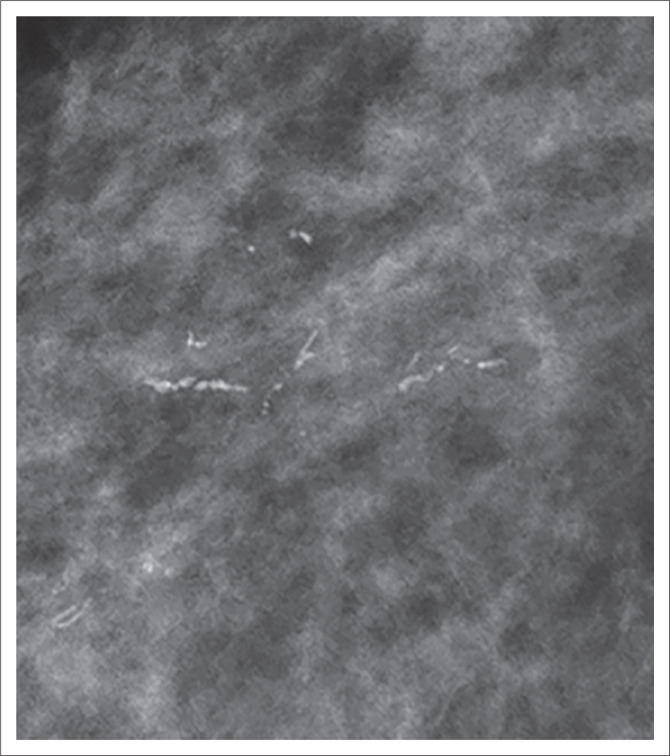
A 63-year-old asymptomatic woman with ductal carcinoma in situ grade II. The mammography image shows linear ductal calcifications.

**FIGURE 2:**
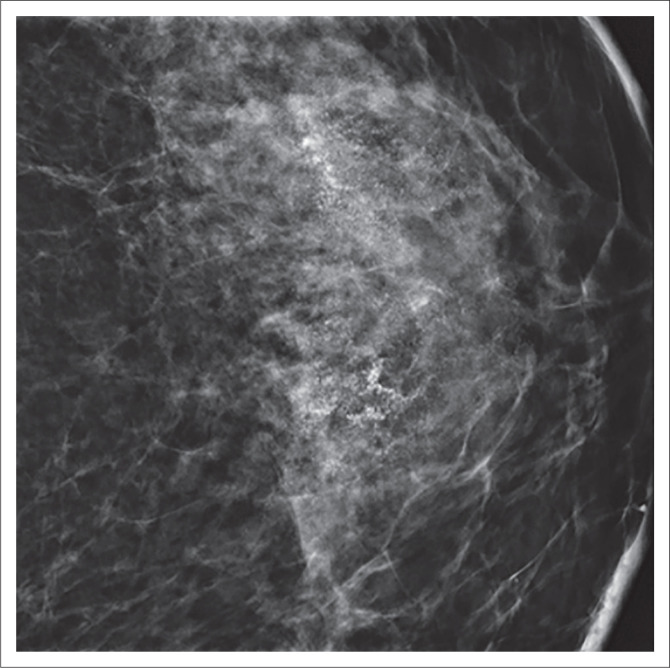
A 51-year-old woman with ductal carcinoma in situ grade III presented with breast pain. The mammography image shows a segmental distribution of amorphous calcifications.

**FIGURE 3:**
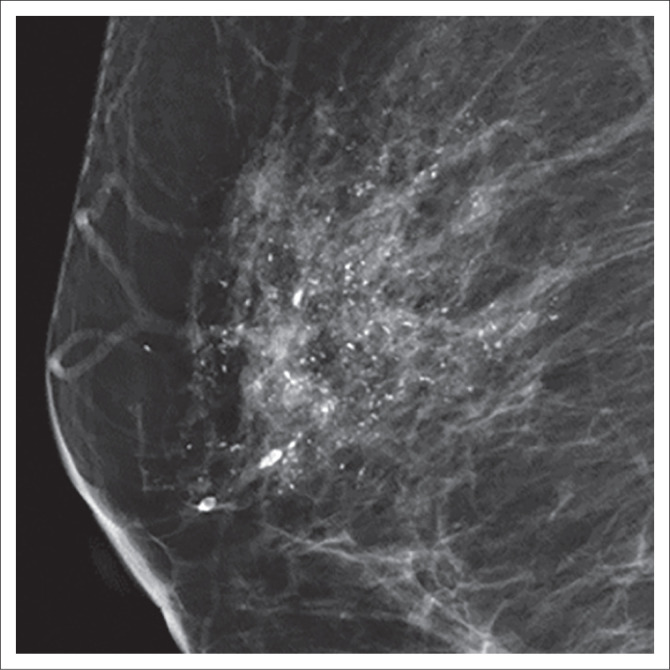
A 49-year-old woman with sclerosing adenosis presented with breast pain. The mammography image shows a segmental distribution of coarse heterogeneous calcifications.

**FIGURE 4:**
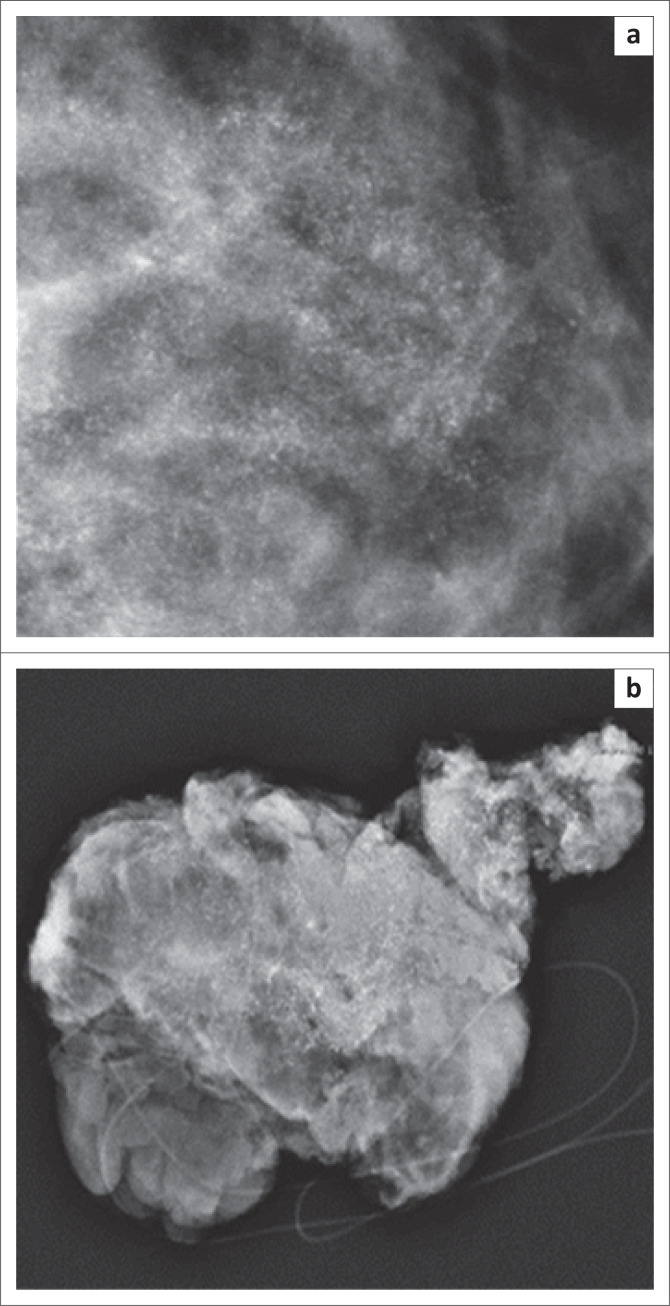
A 49-year-old woman with sclerosing adenosis presented with breast pain. (a) The mammography image shows a regional distribution of amorphous calcifications. (b) Mammography image of breast tissue after surgical excision under wire localisation of the same patient.

Comparison of the morphology and distribution of suspicious calcifications among DCIS and benign breast disease is demonstrated in Table 2. Fine linear calcifications (Figure 1) were more frequent in DCIS than benign breast disease (6/30 [20%] vs 1/71 [1.4%], respectively). The segmental distribution of calcification (Figure 2 and Figure 3) was more evident in DCIS than benign breast disease (7/31 [23.3%] vs 12/71 [11.9%], respectively), but the regional distribution of calcification (Figure 4) was twice as common in benign breast disease than DCIS (15/71 [21.1%] vs 3/30 [10%]). Amorphous calcifications and grouped distribution were seen in both DCIS and benign breast disease.

**TABLE 2:** Comparison of mammographic features.

Features	DCIS calcifications (*n* = 30)		Benign calcifications (*n* = 71)		Total (*N* = 101)		*p*
	*n*	%	*n*	%	*n*	%	
**Morphology**	-	-	-	-	-	-	0.002*
Punctate/round	1	3.3	14	19.7	15	14.9	-
Amorphous	10	33.3	34	47.9	44	43.6	-
Coarse heterogeneous	3	10.0	4	5.6	7	6.9	-
Fine pleomorphic	10	33.3	18	25.4	28	27.7	-
Fine linear	6	20.0	1	1.4	7	6.9	-
**Distribution**	-	-	-	-	-	-	0.011*
Diffuse	N/A	N/A	N/A	N/A	N/A	N/A	-
Regional	3	10.0	15	21.1	18	17.8	-
Group	18	60.0	51	71.8	69	68.3	-
Segmental	7	23.3	5	7.0	12	11.9	-
Linear	2	6.7	0	0	2	2.0	-

DCIS, ductal carcinoma in situ; N/A, not applicable.

* , Statistically significant at *p*-value < 0.05 determined by Fisher’s exact test.

Univariate analysis (Table 3), using logistic regression to analyse the correlation between mammographic features and DCIS, showed that the morphology and distribution of calcifications that significantly related with DCIS were linear descriptor (*p* = 0.003) and segmental distribution (*p* = 0.024). The linear calcifications (odd ratios, 85 [95% CI: 4.48, 1576.51]) and segmental distribution (odd ratios, 7 [95% CI: 1.29,37.91]) were more frequent in DCIS than in benign breast disease.

**TABLE 3:** Logistic regression analysis for predicting ductal carcinoma in situ.

Features	Univariable analysis			Multivariable analysis		
	Crude OR	95% CI	*p*	Crude OR	95% CI	*p*
**Morphology**						
Punctate/round	-	1	-	-	1	-
Amorphous	4.12	0.48, 35.27	0.197	3.98	0.45, 34.90	0.213
Coarse heterogeneous	10.5	0.84, 130.66	0.068	9.92	0.76, 128.89	0.080
Fine pleomorphic	7.78	0.89, 68.19	0.064	6.60	0.73, 59.38	0.092
Fine/linear	84.0	4.48, 1576.51	0.003†	51.72	2.61, 1022.89	0.010†
**Distribution**						
Regional	-	1	-	-	1	-
Group	1.90	0.49, 7.31	0.350	1.61	0.38, 6.80	0.518
Segmental	7.00	1.29, 37.91	0.024†	4.10	0.67, 25.12	0.127
Linear	78256803.95	0, Inf	0.991	22347743.18	0, Inf	0.991

OR, odds ratio; CI, confidence interval.

† , Statistically significant at *p*-value < 0.05 determined by logistic regression.

With multivariate analysis (Table 4), fine linear calcifications were still associated with a significantly increased risk of DCIS (odd ratios, 51.72 [95% CI: 2.61, 1022.89], *p*-value of 0.01), but the distribution for predicting DCIS was not statistically significant.

**TABLE 4:** Comparison of extension and growth rate between ductal carcinoma in situ and benign breast disease.

Tumor growth	DCIS calcification (*n* = 17)	Benign calcifications (*n* = 38)	Total (*N* = 55)	*p*
**Previous extension (mm)**	-	-	-	0.192
Median	5.3000	6.70	5.9	-
IQR	4, 7.8	3.4, 15.30	3.5, 10.8	-
Range	0–33.25	0–38.84	-	-
**Last extension (mm)**	-	-	-	0.920
Median	11	10.3	10.3	-
IQR	8, 14.9	6, 18	6.6, 17.3	-
Range	5.05–36.000	3.19–44.43	-	-
**Growth rate (mm/month)**	-	-	-	0.073
Median	0.5	0.1	0.2	-
IQR	0.2, 0.6	0, 0.4	0.1, 0.6	-
Range	0.019–1.225	−0.012–4.565	-	-

DCIS, ductal carcinoma in situ; IQR, interquartile ranges.

For growth rate comparison, 55 cases had available prior digital mammography images, 17 for DCIS and 38 for benign breast disease. The interval between the previous and latest mammography before mammography-guided wire localisation with surgical excision ranged from 2 months to 64 months. The growth rate of DCIS calcifications was faster than benign calcifications without a statistically significant difference (median, 0.5 mm/month [interquartile ranges {IQR}, 0.2–0.6 mm/month] vs 0.1 mm/month [IQR, 0–0.4 mm/month]; *p* = 0.073) (Table 4).

## Discussion

In several reports over the last four decades, DCIS is considered to be an immediate precursor to potentially lethal invasive breast cancer.^2,5^ Although not all patients diagnosed with breast DCIS will progress to invasive breast cancer, many studies^5,18,19,20,21^ report that about 20% – 50% of untreated DCIS patients were finally detected with invasive breast cancer after over a 10-year period. The differentiation of suspicious calcifications between DCIS and benign breast disease is clinically important for early diagnosis and treatment, resulting in a better prognosis and decreased recurrence rate.^5,22^

According to previous studies, most DCIS cases are frequently detected at screening mammography.^3,4^ The current study also showed that both DCIS and benign breast disease patients were asymptomatic and first noticed on screening examinations. The majority DCIS cases were diagnosed in postmenopausal women older than 50 years.^23^ This study showed a similar result, with the mean patient age of DCIS at 53.5 years. There was a statistically significant correlation between DCIS and postmenopausal status (*p* = 0.01).

Bent CK et al., Burnside ES et al. and Rattanathawornkiti K et al. found that the fine pleomorphic and fine linear morphologies were the first and second mammographic descriptors in DCIS, where the fine linear morphology had the highest positive predictive value (PPV) for malignancy.^11,13,24^ Our results paralleled their studies with a higher incidence of fine linear calcifications in DCIS. This could be supported by the theory that fine linear microcalcifications are located in the terminal ducts of the terminal ductal lobular unit. Calcifications in DCIS occur in the center of the tumor and grow along the mammary duct forming a linear arrangement.^9,25^ Although our data showed that fine pleomorphic calcifications were slightly more frequent in DCIS than in benign breast disease (33.3% vs 25.4%), there was no significantly elevated risk for DCIS (*p* = 0.064). This may be explained by the small sample size in the DCIS group.

Although prior studies found that DCIS could present with punctate or round calcifications, this finding was seen in fewer of our cases than other suspicious calcifications.^26,27^ We found punctate or round calcifications in only one case in the DCIS group with a statistically significantly higher frequency in benign breast disease (*p* = 0.002).

In accordance with current knowledge, a segmental or linear distribution of microcalcifications, known as a ductal distribution, is formed within the adjoining terminal ductal lobular units and tends to occur in DCIS.^13,14^ This study only found a statistically significant correlation with the segmental distribution and DCIS (*p* = 0.024), as noted in previous studies.^11,13,24^ However, these studies also demonstrated that a linear distribution had the highest PPV for malignancy. The incongruous result may be explained by the fact that the linear distribution was rarely detected in both groups (2/30 cases in DCIS and 0/71 cases in benign breast disease), resulting in no statistical significance after statistical analysis. Of note, however, the linear distribution was only observed in DCIS calcifications. We found the regional distribution of calcifications more frequently in benign breast disease than in DCIS (*p* = 0.011). This is related to the fact that these microcalcifications arise in the stromal elements, lobules or glands of the breast and tend to be benign.^13,14^

Beyond microcalcification descriptors, the growth rate of calcifications is considered to be a differentiating factor between DCIS and benign breast disease. A study by Grimm et al found that the growth rate of DCIS microcalcifications was faster than benign breast disease lesions.^28^ Upon analysing the calcification growth rate compared with available prior imaging (55/101), we found that the DCIS calcifications had a faster growth rate than benign calcifications, but this was not statistically significant.

Some DCIS cases were detected and received a tissue diagnosis because of extension of the microcalcifications or microcalcifications that were overlooked on previous imaging. Missed calcifications may be obscured by dense fibroglandular tissue or the calcifications may be too few in number to detect and characterise. Spot magnification views and breast tomosynthesis are appropriate additional techniques to increase the detection of microcalcifications.

### Limitations

The retrospective study design may have introduced selective bias. There was a relatively small sample size (*n* = 101) and an unequal number of patients between the DCIS and benign breast disease groups. Further studies with a larger population and data sets are needed to validate our findings.

## Conclusion

Both DCIS and benign breast disease commonly present with asymptomatic calcifications. Calcification descriptors are considered important elements for early diagnosis and distinguishment of DCIS from other benign breast conditions. Fine linear branching calcifications and segmental distribution were found to correlate with DCIS in this study. Amorphous calcifications and grouped distributions were common features in both DCIS and benign disease, and thorough evaluation with additional mammographic techniques is highly suggested.
